# Supplementary material for: Mitofusin-2 Down-Regulation Predicts Progression of Non-Muscle Invasive Bladder Cancer
Source: Diagnostics (Basel). 2021 Aug 20;11(8):1500. doi: 10.3390/diagnostics11081500 (PMC8394056; doi:10.3390/diagnostics11081500)
Supplement: Supplementary file 1 [file diagnostics-11-01500-s001.zip › diagnostics-1325830-supplementary.pdf]

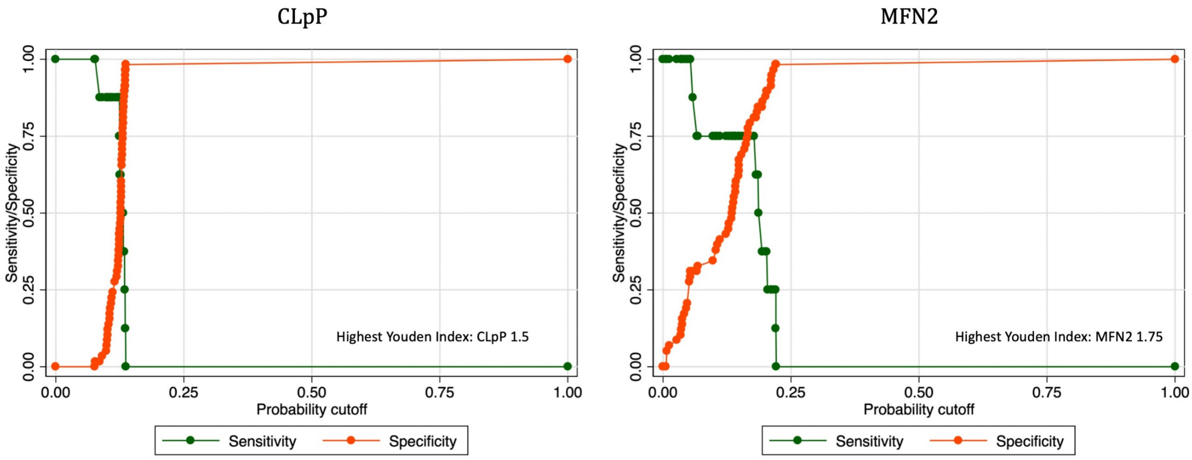

Figure S1. Graphical representation of Sensitivity and specificity according to mitochondrial proteins expression levels in the prediction of disease progression at 24 months of follow-up. The cutoffs of Mfn2 and ClpP with the highest Youden Index and thus, with the best combination of sensitivity and specificity were 1.75 and 1.5 for Mfn-2 and ClpP, respectively.
